# Challenges and pitfalls during CRT implantation in patients with persistent left superior vena cava

**DOI:** 10.1007/s10840-024-01761-7

**Published:** 2024-02-12

**Authors:** Deniz Akdis, Julia Vogler, Malte-Maria Sieren, Nadine Molitor, Tom Sasse, Huong-Lan Phan, Lorenzo Bartoli, Niels Grosse, Ardan M. Saguner, Urs Eriksson, Firat Duru, Daniel Hofer, Alexander Breitenstein, Roland Richard Tilz, Stephan Winnik

**Affiliations:** 1 Department of Cardiology, University Heart Center Zurich, University Hospital Zurich, Zurich, Switzerland; 2 Division of Cardiology, GZO Zurich Regional Health Center Wetzikon, Wetzikon, Switzerland; 3 Department of Rhythmology, University Heart Center Lübeck, University Hospital Schleswig-Holstein, Lübeck, Germany; 4 Department of Radiology, University Hospital Schleswig-Holstein, Lübeck, Germany; 5 Department of Cardiology, Kantonsspital Aarau, Aarau, Switzerland; 6 Institute of Cardiology, Sant’Orsola-Malpighi Hospital, IRCCS, Bologna, Italy; 7 Department of Experimental, Diagnostic and Specialty Medicine-DIMES, University of Bologna, Bologna, Italy; 8 Department of Cardiology, Triemli Hospital, Zurich, Switzerland; 9 German Center for Cardiovascular Research (DZHK), Partner Site Hamburg/Kiel/Lübeck, Lübeck, Germany

**Keywords:** Cardiac resynchronization therapy, Persistent left superior vena cava, Pacemaker, Defibrillator

## Abstract

**Background:**

Persistent left superior vena cava (PLSVC) is a rare venous anomaly, affecting 0.3–0.5% of the general population. Cardiac resynchronization therapy (CRT) implantation in patients with PLSVC is challenging due to a complex anatomy. Moreover, data on CRT implantation in this patient population is scarce. Our aim was to report a series of patients with PLSVC and CRT implantation focusing on challenges and pitfalls.

**Methods:**

Electronic medical databases on patients with CRT implantation at the University Heart Centers in Zurich, Switzerland, and Lübeck, Germany, were screened for individuals with a PLSVC. Clinical and demographic characteristics as well as procedural data were reported in all patients.

**Results:**

This study presents six cases with a median age of 66 years. CRT implantation was successful in five patients, leading to a reduced QRS duration and improved left ventricular ejection fraction. Atrial fibrillation, ischemic cardiomyopathy, valvular heart disease, and dilated cardiomyopathy were observed in this group as underlying conditions. Specialized tools, such as active fixation left ventricular leads, were utilized. One patient experienced major complications.

**Conclusions:**

This case series shows that although challenging, conventional endovascular CRT implantation is feasible in PLSVC patients. Specialized tools for visualization and fixation may help. Our experiences highlight the importance of preprocedural evaluation of the anatomy and precise intervention planning.

**Graphical Abstract:**

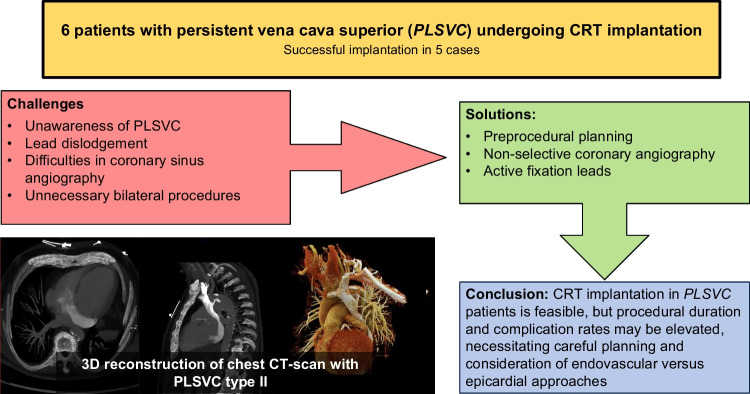

**Supplementary Information:**

The online version contains supplementary material available at 10.1007/s10840-024-01761-7.

## Introduction

Persistent left superior vena cava (PLSVC) is a congenital anomaly with a prevalence of around 0.5% in patients undergoing device implantation [1]. The PLSVC commonly drains into the coronary sinus (CS), rarely into the left atrium, and is usually asymptomatic and an incidental finding. However, it can manifest through supraventricular and ventricular arrhythmias or may be associated with other congenital anomalies [2]. The most frequently used classification by Schummer et al. [3] distinguishes between three types of the thoracic venous system: type I refers to a normal venous anatomy, type II describes a PLSVC with absent right superior vena cava, and type III describes the presence of both superior venae cavae, either connected through an innominate vein (type III A) or without a connection (type III B). If a PLSVC is not diagnosed before the implantation of a cardiac resynchronization therapy (CRT) device, it can result in incorrect lead positioning and complications. This is especially critical in patients with a PLSVC type II and III B, where the CS can be massively dilated. Echocardiography may reveal an enlarged CS, suggestive of the presence of a PLSVC. Nevertheless, elevated filling pressures in the heart failure population may also lead to a dilated CS. Positioning of transvenous leads, especially CS leads, is challenging in patients with PLSVC since a proper assessment of coronary venous anatomy using retrograde venography is limited, and identifying target veins is often difficult by antegrade venography. Moreover, lead manipulation and stability are impaired due to the dilation of the coronary venous system [3, 4]. Therefore different types of leads might be necessary for adequate placement, and, in some cases, active fixation may be required [5]. Here, we present the experience of two different European tertiary referral hospitals with CRT-pacemaker (CRT-P) and CRT-defibrillator (CRT-D) implantation in patients with PLSVC, highlighting different approaches with their associated challenges and pitfalls.

## Methods

All patients undergoing CRT implantation or upgrade between 02/2018 and 05/2023 at both participating centers (University Heart Center Lübeck, Germany, and University Heart Center Zurich, Switzerland) were reviewed using electronic medical databases, and patients with PLSVC were identified. The study complied with the local ethical guidelines, and prospectively included patients gave their informed consent. Clinical and demographic baseline characteristics, multiple 12-lead surface ECGs, imaging findings, as well as procedural data were available in all patients. We obtained standard echocardiographic measurements of cardiac chambers and ventricular function according to current guidelines [6].

## Results

### Case descriptions

#### Case # 1

**Fig. 1:**
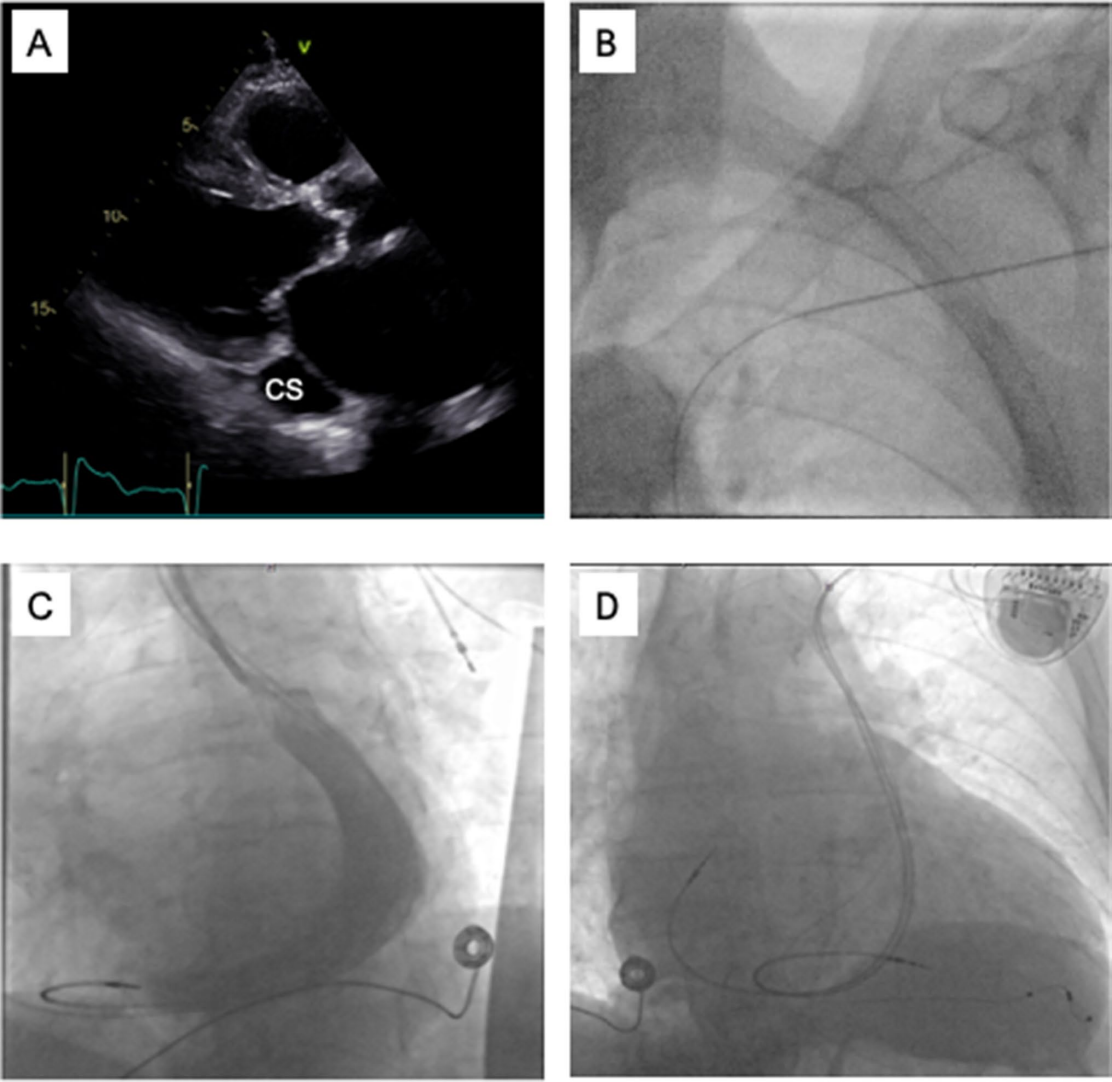
Case # 1: transthoracic echocardiography showing a dilated coronary sinus (CS) in parasternal long axis view (**A**). Wire passing through PLSVC (**B**). Non-selective antegrade CS angiography showing dilated CS and the right ventricular lead, left anterior oblique view (**C**). Fluoroscopic overview after implantation of all three leads and CRT-P device, postero-anterior view (**D**)

A 76-year-old patient with coronary artery disease, aortic stenosis, and a left ventricular ejection fraction (LVEF) of 22% presented for atrioventricular-node ablation due to permanent symptomatic atrial fibrillation and insufficient rate control of paroxysmal tachycardic intrinsic conduction. In accordance with current guidelines, the patient was scheduled for biventricular pacing to avoid right ventricular-only pacing [6, 7]. The patient did not wish to receive a defibrillator; therefore, the implantation of a CRT-P was scheduled. Since echocardiography revealed a dilated coronary sinus, PLSVC was suspected (Fig. 1A). A left-sided venogram via the left cubital vein confirmed the presence of a PLSVC with a missing innominate vein (type III B). After left-sided incision and preparation, three guidewires were advanced through the PLSVC into the coronary sinus and into the right atrium (Fig. 1B). Next, atrial and ventricular leads were delivered. The ventricular lead was looped once in the right atrium to pass the tricuspid valve and was placed at the inferior midventricular septum, obtaining good electrical parameters. Using the Attain Command™ guiding catheter (Medtronic) to canulate the coronary sinus, a non-selective, antegrade occlusion venogram showed the dilated CS without any hints of a target vein (Fig. 1C). However, direct injection of contrast medium over an Attain Select™ II + SureValve 130° sub-selection catheter (Medtronic) revealed a posterolateral vein. Targeted cannulation of the posterolateral vein was achieved using the sub-selection catheter and a PT2™ Moderate Support Guidewire (Boston Scientific). The lead was then placed in the posterolateral vein using an over-the-wire technique. An S-shaped, passive fixation electrode was used (Medtronic Attain Performa 4598). Acceptable electrical parameters were acquired without phrenic nerve capture. Guiding catheters were removed, the leads and the CRT device were fixed inside the epifascial pocket, and the final fluoroscopy confirmed correct lead position of all 3 leads (Fig. 1D) before wound closure. The total procedural time was 200 min. No periprocedural complications occurred, and subsequent chest X-rays verified optimal and stable lead positions. The QRS duration with 99% biventricular pacing was 130 ms (QRS duration during RV-pacing was 180 ms). Follow-up after 1 month confirmed stable electrical parameters, 99% biventricular stimulation, and good wound healing. Under adequate beta-blocker therapy, the underlying rhythm remained an atrial fibrillation with a heart rate of around 45/min. Therefore, atrioventricular-nodal ablation was no longer needed.

#### *Case* # *2*

A 65-year-old patient with Long-QT-Syndrome and high-degree AV block received a dual-chamber pacemaker (right-sided implantation) in 2007. After episodes of Torsades de Pointes, an implantable cardioverter defibrillator (ICD) was implanted in 2015, and the old right ventricular lead was abandoned. Subsequently, noise episodes were detected both on the atrial and the ventricular lead. Therefore, an extraction was scheduled. Due to the high proportion of right ventricular pacing (> 99%) and a mildly reduced LVEF of 49%, lead extraction and an upgrade to a CRT-D was planned [6, 7]. Intrinsic QRS duration was 150 ms, and QRS duration with RV-pacing was 180 ms.

**Fig. 2:**
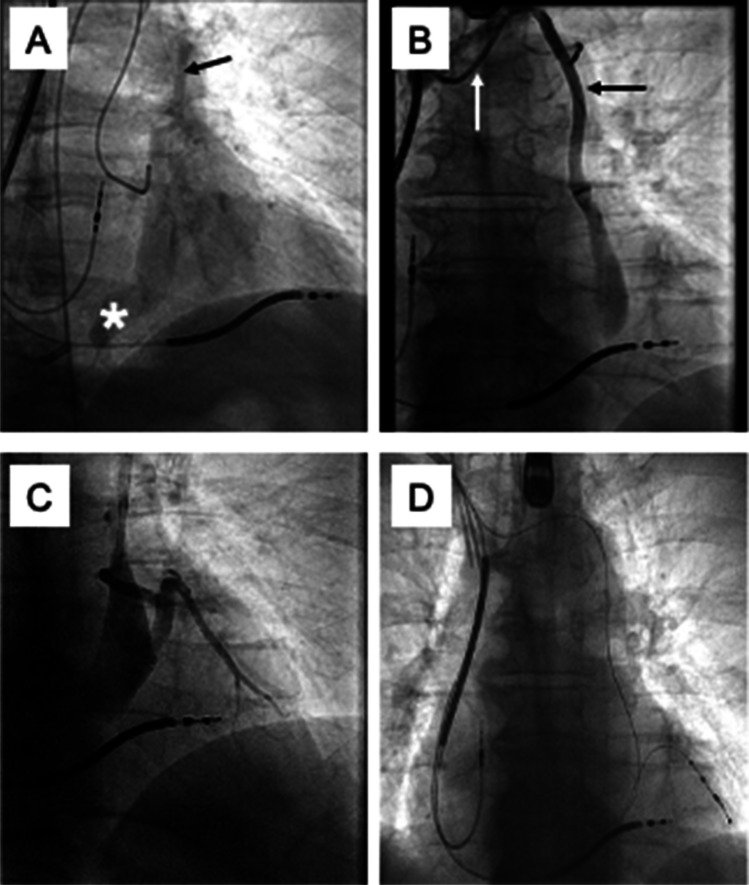
Case # 2: late phase of the coronary angiogram through femoral arterial access revealing a markedly dilated coronary sinus, without drainage into the right atrium due to coronary sinus ostial atresia (white asterisk) and a PLSVC (black arrow), postero-anterior (**A**). Antegrade venogram demonstrating an innominate vein (white arrow) as well as the PLSVC (black arrow), postero-anterior (**B**). Antegrade venogram in the CS identifying a suitable lateral vein, postero-anterior (**C**). Fluoroscopic overview after implantation of all three leads, postero-anterior (**D**)

The procedure was performed under general anesthesia. First, bilateral venous femoral access and left-sided arterial femoral access were established. Right-sided venogram via cubital venous access showed an occlusion of the venous system proximal to the right subclavian vein. Hence, venous access of the extraction site was to be maintained and used for CRT re-implantation. The old, right-sided device, as well as the leads were relieved from adhesions and detached. Extraction was successfully performed using a Spectranetics Sub-C and a Spectranetics TightRail™ tool (Philipps). Three guidewires were inserted using the primarily inserted extraction sheaths. The right ventricular lead was implanted at a mid-septal position, while the atrial lead was positioned in the right atrial appendage. Using conventional sheaths and catheters (Biosense Webster steerable decapolar catheter and Medtronic steerable sheath), the cannulation of the coronary sinus ostium was unsuccessful. Therefore, a coronary angiogram was performed through femoral arterial access, and a markedly dilated coronary sinus and a PLSVC draining into the coronary sinus were visualized, suggesting a type III PLSVC (Fig. 2A). The coronary sinus did not drain into the right atrium but into the PLSVC, likely due to a CS ostial atresia. An additional venogram revealed an innominate vein (Type III A) (Fig. 2B). A stiff Terumo-Wire could be placed through the innominate vein into the PLSVC, and a sub-selection catheter (Attain Select™ II + SureValve 130°, Medtronic) was advanced and placed into the coronary sinus. An antegrade venogram identified a suitable lateral vein (Fig. 2C), and the sub-selection catheter was subsequently inserted into the target vessel. Over a PT2™ Moderate Support Guidewire (Boston Scientific), an S-shaped quadripolar CS-lead was placed in the target vein. Adequate electrical parameters were measured without phrenic nerve capture. Final fluoroscopy confirmed correct lead positions (Fig. 2D). All sheaths and cannulation catheters were removed, the electrodes and the CRT device were fixed, and the wound was closed.

The total procedural time was 180 min. No periprocedural complications occurred, and serial fluoroscopy confirmed an optimal lead position. Stimulated QRS duration was 125 ms, and post-procedural echocardiography showed no worsening of tricuspid regurgitation. Follow-up after 1 month revealed stable electrical parameters, 99% biventricular pacing and good wound healing.

#### *Case* # *3*

**Fig. 3:**
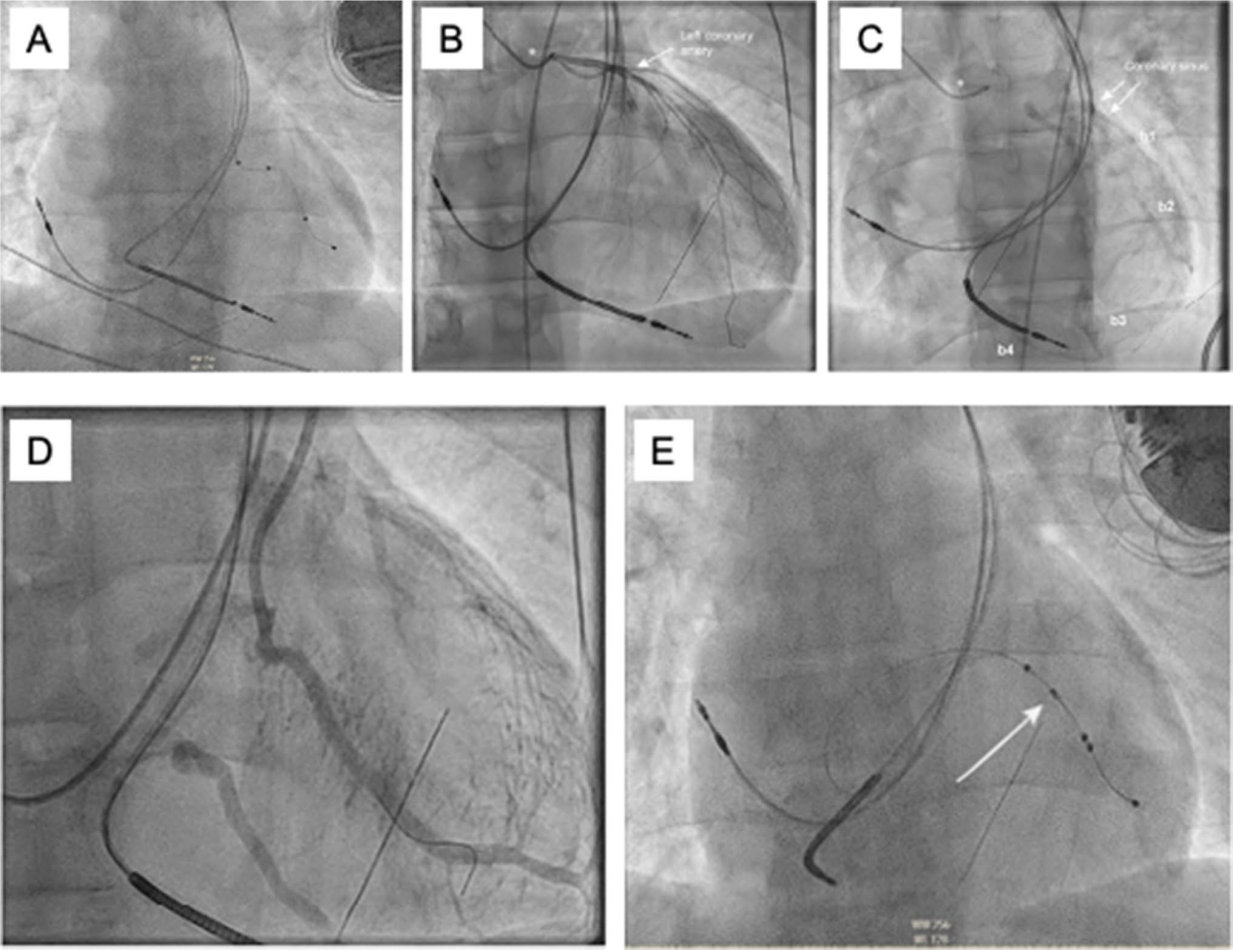
Case # 3: initial position of the passive fixation CS lead, postero-anterior view (**A**). Coronary angiography during the revision following CS lead dislodgement, showing the left coronary artery and the Judkins left catheter (white asterisk), right anterior oblique (**B**) Venous phase of the coronary angiography showing the CS with different branches (b1–b4) and the Judkins left catheter (white asterisk), right anterior oblique (**C**). Direct venous angiography of the posterolateral CS target vein during the second procedure, postero-anterior (**D**). Final position of the active fixation lead (Medtronic Attain Stability.™), the white arrow points to the active fixation screw of the lead, left anterior oblique (**E**)

A 35-year-old patient with dilated cardiomyopathy (DCM) of unknown origin, LBBB (QRS 155 ms) and reduced LVEF (30%) on optimal medical therapy (OMT) was admitted for elective CRT-D implantation. After left-sided skin incision and pocket preparation, cephalic vein cutdown (1 wire) and double subclavian vein puncture were performed. The course of the guide wires revealed PLSVC. Antegrade coronary sinus angiography was performed using a sub-selection catheter and an occlusion balloon, which revealed a posterolateral target vein. After one failed attempt to implant the CS lead, the right ventricular ICD and the right atrial leads were implanted. Afterwards, the target coronary sinus branch was re-cannulated using a sub-selection catheter (90°, Abbott) and a Guidant Pilot 50 wire. A quadripolar CS-lead was implanted in over-the-wire technique with good electrical parameters and no phrenic nerve capture (Fig. 3A). All catheters were removed. The QRS duration under biventricular pacing was 98 ms. The total procedure duration was 167 min. However, on the first postoperative day, dislodgement of the CS lead with exit block in all configurations occurred. A second procedure was scheduled. After having difficulties re-cannulating the target vein, a diagnostic coronary angiography via femoral access was performed. The venous phase revealed the entry site of the desired target vein (Fig. 3B, C). Another direct venous angiography revealed the target vein (Fig. 3D). A new active fixation quadripolar lead (Medtronic Attain Stability™ Quad) was successfully implanted (Fig. 3E). The procedure duration was 148 min. Nevertheless, the following night, the patient was resuscitated due to ventricular fibrillation (VF). Device interrogation was suggestive of RV exit block due to a macro dislodgement of the right ventricular lead. During a third and final procedure, the RV lead was successfully repositioned. No further complications occurred. The patient was discharged without any sequelae. During follow-up, electrical parameters remained stable, and the LVEF improved to 48% with 98% biventricular stimulation.

#### *Case* # *4*

An upgrade from a dual chamber ICD to a CRT-D was planned in a 64-year-old patient with DCM, an LVEF of 30%, permanent RV pacing, and symptomatic heart failure (NYHA III). The patient was known for a PLSVC. No preoperative angiography was performed. He had already received a right-sided dual chamber ICD after a failed left-sided implantation due to a type III PLSVC following resuscitation for VF in an external hospital. The CS was cannulated with a Medtronic Attain Command™ 6250 V-MPR catheter and a diagnostic AL2 catheter from the right side. A bipolar, passive fixation lead was successfully implanted in a small posterolateral branch. The procedure duration was 102 min. Electrical parameters remained stable until discharge.

#### *Case* # *5*

**Fig. 4:**
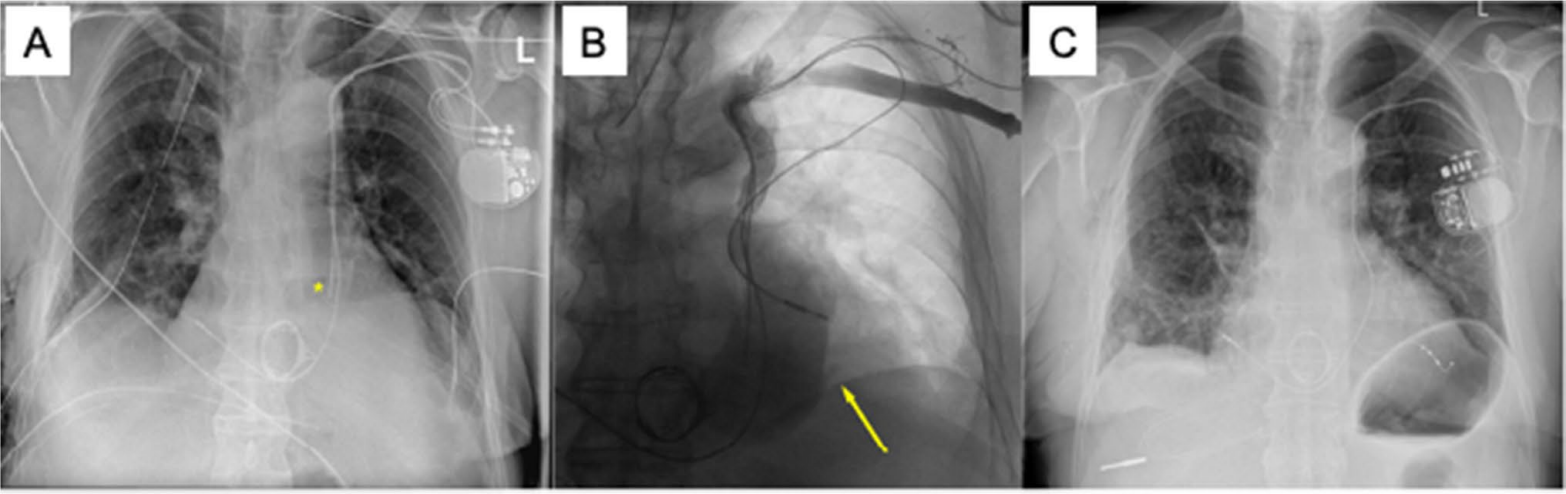
Case # 5: chest X-ray after tricuspid valve replacement with the asterisk at the end of the stripped-down right ventricular lead (**A**). Initial angiography showing PLSVC and the ostium of a posterolateral CS branch (yellow arrow) (**B**). Final chest X-ray on first postoperative day (**C**)

A 72-year-old patient with known PLSVC and a left-sided dual-chamber pacemaker implanted in 2012 underwent cardiac surgery for torrential tricuspid regurgitation. During surgical tricuspid valve replacement, the right ventricular lead was cut secondary to severe adhesions (Fig. 4A). The patient had a long device history, with initial left-sided implantation of a dual-chamber pacemaker for sinus node dysfunction in 1998, lead extraction, and right-sided reimplantation, followed by another device extraction and left-sided reimplantation in 2012, each due to device infections. During the disease course, he became more dependent on atrial as well as ventricular pacing. To avoid additional cardiothoracic surgery and damage to the newly replaced tricuspid valve, an endovascular approach with the implantation of a CS lead, instead of another transvalvular right ventricular lead, was planned. The procedure was performed under general anesthesia. The periprocedural angiography of the left-sided brachiocephalic vein showed a patent subclavian vein, the PLSVC, and a posterolateral coronary sinus target branch (Fig. 4B). The stripped-down right ventricular lead was successfully extracted using a lead locking stylet (Spectranectics, LLD EZ) and a nine French Tightrail™ (Spectranectis/Philips), while the intact right atrial lead was preserved. After subclavian vein puncture, a Medtronic Attain Command™ MB2 coronary sinus cannulation catheter and a 135° sub-selection catheter (Attain Select II™) were advanced through the PLSVC, and the posterolateral target branch was cannulated. Given the high risk of LV lead dislodgement in a pacemaker-dependent patient, an active fixation quadripolar LV lead was implanted (Medtronic Attain Stability™ Quad). Sufficient electrical parameters were achieved without phrenic nerve capture. A Biotronik Enitra 8 HF-T QP device was chosen, and an IS1 pin was plugged into the RV port of the device (Fig. 4C). The procedure was completed in 104 min. Electrical parameters remained stable until discharge.

#### Case # 6 (unsuccessful CRT-implantation)

**Fig. 5:**
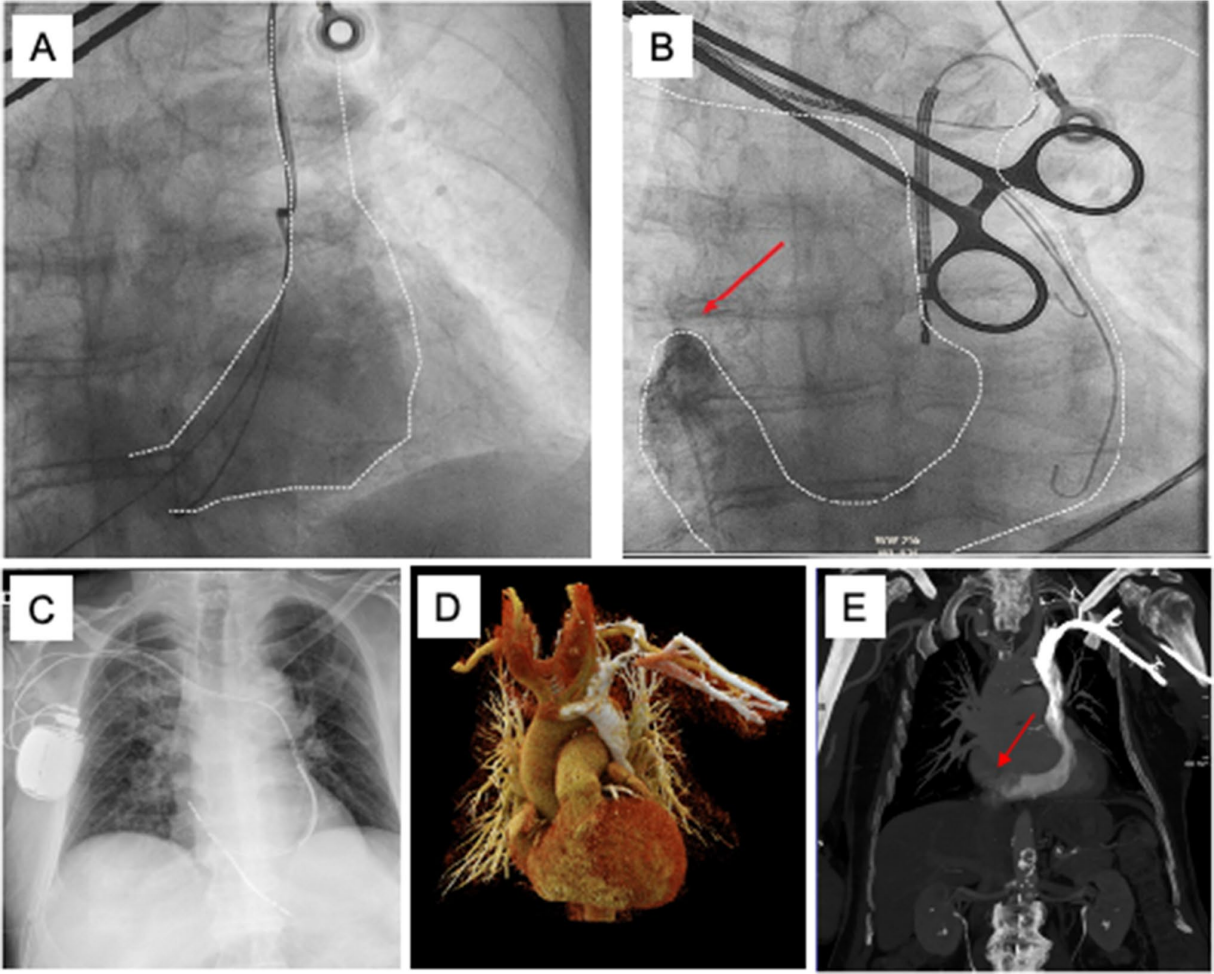
Case # 6: initial non-selective angiography showing a largely dilated CS outlined by the dotted lines, postero-anterior (**A**). Venous angiography via femoral access confirming the presence of an isolated PLSVC and showing the dilated CS (dotted lines and red arrow). Guidewires and RV-lead were inserted from the right side and are also visible, postero-anterior (**B**). Chest X-ray after dual chamber ICD implantation (**C**). 3D reconstruction of chest CT-scan showing the venous anomaly (PLSVC type II) (**D**). Conventional chest CT-scan with the missing right-sided superior vena cava (red arrow) (**E**)

An 81-year-old patient with known coronary artery disease and severely reduced LVEF (20%) presented with syncope, highly suspicious for an arrhythmic origin. After excluding acute ischemia, the decision was made to implant a CRT-D since the patient also suffered from an LBBB with a QRS duration of 160 ms. After left-sided double subclavian vein puncture, the course of the guide wires and non-selective venous angiography revealed PLSVC with a massively dilated coronary sinus (Fig. 5A). In anticipation of a type III thoracic venous anatomy and consideration of the anticipated prolonged procedure duration in an old and frail patient, the operators decided to perform a right-sided implantation without a preceding venogram. After right-sided cephalic cut-down, both guide wires still went into the CS via the PLSVC, suggestive of type II thoracic venous system with an isolated PLSVC and absent right-sided superior vena cava. Venous angiography via femoral venous access using a Judkins right catheter confirmed the diagnosis (Fig. 5B). To avoid an excessively long procedure time, the operators opted against a CS lead implantation and implanted a dual chamber ICD using a dual coil defibrillation electrode, considering both the right-sided device location and the atypical course of the RV lead through the coronary sinus (Fig. 5C). The total procedure duration was 136 min. A chest computed tomography (CT) scan of the patient that was subsequently available once again confirmed the venous anomaly of an isolated PLSVC (type II) with an absent right superior vena cava (Fig. 5D, E).

### Clinical data and procedure outcomes

All patients in this study were male, with a median age of 66 years. Median NYHA class was II. Three patients (50%) had atrial fibrillation, two (33%) had ischemic cardiomyopathy, two (33%) had valvular heart disease, and two (33%) suffered from dilated cardiomyopathy. All patients were on optimal medical therapy for heart failure (Table 1). Median procedure duration was 152 min, and median fluoroscopy time was 25 min. Median left ventricular sensing was 18 mV, while median left ventricular threshold was 1.1 V with a pulse duration of 0.5 ms. Follow-up data were available in all patients, in whom a CRT device was implanted, with a median follow-up of 5 months. In these patients, QRS duration could be reduced from a median of 150 ms before to a median of 130 ms after CRT implantation. LV function improved from a median of 30 to 45%. One patient suffered major complications (case #3): lead dislocation with subsequent cardiopulmonary resuscitation due to VF. The same patient underwent CS lead revision, complicated by a pneumothorax.

**Table 1:** Baseline characteristics

Demographics	
Age, years, median (IQR)	69 (11)
Male, *n* (%)	6 (100)
BMI, kg/m^2^, median (IQR)	25 (3)
NYHA class, median (IQR)	2 (1)
Underlying disease	
Atrial fibrillation, *n* (%)	3 (50)
Ischemic cardiomyopathy, *n* (%)	2 (33)
Valvular heart disease, *n* (%)	2 (33)
Dilated cardiomyopathy, *n* (%)	2 (33)
Hypertension, *n* (%)	4 (66)
Medical therapy	
Betablocker, *n* (%)	6 (100)
ACEi/ARNI, *n* (%)	6 (100)
MRA, *n* (%)	3 (50)
SGLT2i, *n* (%)	4 (66)
Loop diuretic, *n* (%)	4 (66)
Serum levels of natriuretic peptides	
Baseline NTproBNP, pg/mL, median (IQR)	400 (230)

Number of patients *n* = 6; data are presented as median and interquartile range (IQR).

Table 2 summarizes our individual key findings of patients who received a resynchronization system, and Supplementary Table 1 shows the combined data from all patients.

**Table 2:** Individual procedural and follow-up data

Case	PLSVC type	Targeted CS-branch	CS-lead	Implanted device	Procedure duration [min]	Fluoroscopy time [min]	LV sensing [mV]	LV treshold [V]	LVEF before [%]	LVEF after [%]	QRS before [ms]	QRS after [ms]
# 1	III B	Posterolateral	Quadripolar passive fixation	CRT-P	200	46	4.6	2	22	22	145	130
# 2	III A	Lateral	Quadripolar passive fixation	CRT-D	180	30	16	0.75	49	54	150	125
# 3	III B	Posterolateral	Quadripolar active fixation	CRT-D	167	46	20	0.8	30	48	155	98
# 4	III A	Posterolateral	Bipolar passive fixation	CRT-D	102	11	Paced	1.7	30	38	150	130
# 5	III A	Posterolateral	Quadripolar active fixation	CRT-P	104	14	20	1.1	52	52	150	145

Number of patients *n* = 5; only including patients who received a resynchronization system. LV threshold was measured with a pulse duration of 0.5 ms.

## Discussion

Our study presents one of the largest and most comprehensive case series of patients with various types of PLSVC undergoing CRT implantation. The feasibility of left-sided CRT implantation in PLSVC has only been shown in a few cases before [4, 5, 8–11]. A right-sided approach or epicardial lead placement is often chosen in daily clinical routine, if possible, because of the inability or insecurity to perform successful left-sided implantation. Our series of real-world PLSVC cases with different anatomies (type II, IIIa, IIIb) demonstrated successful CRT implantation in five out of six cases using conventional CS cannulation and sub-selection catheters of different vendors. CS lead placement was omitted in one patient with type II PLSVC due to frailty.

The procedures were conducted within a reasonable duration, with a median procedure time of 152 min, yet with higher radiation exposure (median 1200 cGy*cm^2^). However, the rate of complications associated with CRT implantation in patients with PLSVC were not significantly elevated when compared to historical data from patients with normal venous anatomy undergoing CRT [12, 13].

Nevertheless, our cases also underline that significant challenges and complications were associated with the unawareness of the presence and/or different types of this venous anomaly, such as the difficulty in performing coronary sinus and side branch angiography, the inability to conduct retrograde occlusion coronary sinus angiography, the struggle to secure a stable coronary sinus lead position/lead dislodgement, and the necessity for bilateral procedures.

The key challenges were addressed using different strategies. In case #2, a non-selective coronary angiography helped visualize the dilated coronary sinus and the PLSVC during the late/venous phase. As previous procedures in this patient were done from the right side, the presence of a PLSVC was unknown prior to intervention. The angiography also revealed a CS ostial atresia, a rare anomaly that can lead to failure of coronary sinus cannulation [11, 14]. Since these cases are often associated with coronary sinus drainage via a persistent left-sided superior vena cava that connects through an innominate vein to a right-sided superior vena cava (type IIIb), a right-sided approach is possible, as in our case. In case #3, an indirect (late/venous) CS angiography helped identify the CS itself and possible target veins. Usually, none of the available balloon catheters are sufficiently large to perform an occlusion angiography of the dilated CS. Moreover, antegrade cannulation with persistent venous run-off necessitates selective venous angiography of potential target veins using sub-selection catheters. These also support successful CS lead delivery.

Active fixation leads may be necessary to avoid lead dislocation in dilated target veins, a recurrent complication in PLSVC patients, as illustrated by case #3. In this case and case #5, choosing an active fixation lead was the key to success. Thus, the presence of a PLSVC may constitute a clear indication for an active fixation lead (currently only available from Medtronic). Nonetheless, case-based decisions with weighing of pro’s and con’s are necessary, and although studies have demonstrated that the active fixation LV-lead is safe and associated with a lower rate of lead displacement, difficulties during extraction in cases of a rising threshold or infections can occur, and further investigations focusing on long-term extractability are necessary [15].

Two patients underwent a bilateral approach. This resulted in a prolonged procedure duration, bilateral incision, and venous puncture. A bilateral approach also increases the risk of periprocedural complications. Especially in older or frail patients, this must be taken into consideration in advance. Therefore, a bilateral peripheral venography should be performed before changing sides intraoperatively. Once a PLSVC is diagnosed during a left-sided implantation procedure, the next best step is to perform a venous angiography through the right internal jugular vein, allowing to differentiate between type II and type III PLSVC.

As illustrated above, the main problems in patients with PLSVC undergoing pacemaker, ICD, or CRT implantation is the preoperative unawareness of this rare anomaly, as patients are usually asymptomatic. A PLSVC can be either diagnosed by bilateral venography (gold standard), a chest CT scan as shown in case #6 or a bilateral contrast echocardiography (“bubble study”) with the injection of agitated saline from both the left and the right peripheral veins [16]. One of the distinct features of the presence of a PLSVC is a dilated CS. Therefore, through echocardiography, it is possible to suspect the presence of a PLSVC. Although a dilated CS may manifest in various other pathologies, it is crucial that operators pay attention to this feature in a preoperative transthoracic echocardiography and initiate further investigations, as mentioned above, in order to confirm the diagnosis and document the thoracic venous anatomy. It is also possible to perform an additional coronary computed tomography venography to identify the optimal CS branch to target, as previously demonstrated [17, 18].

In case of difficulties in transvenous positioning or if difficult anatomies are revealed during preprocedural planning, surgical techniques should be considered as well. Although the epicardial approach can necessitate a median or lateral thoracotomy, minimally invasive thoracotomies or complete thoracoscopic methods exist and are gaining increasing importance [19, 20].

This real-world case series has to be interpreted in light of the following limitations: we present purely observational data from a small number of patients. Our clinical information was mostly gathered retrospectively from regular visits. Therefore, long-term follow-up data were not available in all patients.

Nevertheless, this case series is among the largest series of CRT in PLSVC to date, and we provide comprehensive data, demonstrating that meticulous perioperative evaluation and awareness of thoracic venous anomalies allows successful CRT implantation and/or lead extraction, irrespective of the implantation side. Further research involving larger cohorts is imperative to refine and validate the optimal techniques and approaches for optimizing outcomes in PLSVC patients undergoing CRT interventions.

## Conclusion

Our findings emphasize the potential challenges encountered when performing endovascular cardiac resynchronization therapy (CRT) implantation in this unique patient population. While conventional endovascular CRT implantation, whether from the left or right side, is feasible in patients with PLSVC, it is essential to note that the procedure duration and complication rates can be elevated due to the challenging anatomy. Consequently, meticulous periprocedural planning and the utilization of specialized visualization tools and active fixation leads can offer significant advantages. Furthermore, individual risks associated with an endovascular versus epicardial approach have to be considered, and in cases where a PLSVC and a right-sided superior vena cava coexist, a right-sided approach might be preferable.

## Supplementary Information

Below is the link to the electronic supplementary material.

Supplementary file1 (DOCX 18 KB)

## Data Availability

The data that support the findings of this study are available from the corresponding author, [S.W.], upon reasonable request.

## Abbreviations

CRT: Cardiac resynchronization therapy
CRT-P: Cardiac resynchronization therapy-pacemaker
CRT-D: Cardiac resynchronization therapy-defibrillator
CS: Coronary sinus
DCM: Dilated cardiomyopathy
ICD: Implantable cardioverter defibrillator
PLSVC: Persistent left superior vena cava
LBBB: Left bundle branch block
LVEF: Left ventricular ejection fraction
OMT: Optimal medical therapy
LV: Left ventricle
RV: Right ventricle
BMI: Body mass index
NYHA: New York Heart Association
ACEi: Angiotensin-converting enzyme inhibitor
ARNI: Angiotensin receptor/neprilysin inhibitor
MRA: Mineralocorticoid receptor antagonists
SGLT2i: Sodium glucose linked transporter 2 inhibitor
NTproBNP: N-terminal pro-B-type natriuretic peptide
VF: Ventricular fibrillation
