# Supplementary material for: Challenges and pitfalls during CRT implantation in patients with persistent left superior vena cava
Source: J Interv Card Electrophysiol. 2024 Feb 12;67(7):1505–16. doi: 10.1007/s10840-024-01761-7 (PMC11522099; doi:10.1007/s10840-024-01761-7)
Supplement: Supplementary file 1 — Supplementary file1 (DOCX 18 KB) [file 10840_2024_1761_MOESM1_ESM.docx]

**Supplementary data**

**Table 1: Procedural and follow-up data**

| **Procedural data** |  |
| --- | --- |
| Procedure duration [min], median (IQR) | 152 (76) |
| Fluoroscopy time [min], median (IQR) | 25 (32) |
| Area dose product [cGy*cm^2^], median (IQR) | 1200 (3249) |
| **LV-lead measurements** |  |
| LV sensing, mV, median (IQR)* | 18 (10) |
| LV threshold, V, median (IQR)* | 1.1 (1.1) |
| **Follow-up data** |  |
| Baseline QRS-duration [ms], median (IQR)* | 150 (5) |
| QRS- duration after CRT [ms], median (IQR)* | 130 (26) |
| Complications, n (%)* | 1 (16) |
| LVEF baseline [%], median (IQR)* | 30 (25) |
| LVEF follow-up [%], median (IQR)* | 43 (21) |

Number of patients n = 6 (follow- up n = 5), data are presented as median and interquartile range (IQR).

* Only including patients who received a resynchronization system.
